# MEMORY FOR PICTURES AND WORDS AFTER A NATURAL DISASTER

**DOI:** 10.1093/geroni/igz038.1032

**Published:** 2019-11-08

**Authors:** Katie E Cherry, Katelyn McKneely, Quyen Nguyen, Shui Yu, Laura Sampson, Sandro Galea, Matthew R Calamia, Emily M Elliott

**Affiliations:** 1 Louisiana State University, Baton Rouge, Louisiana, United States; 2 Boston University, Boston, Massachusetts, United States; 3 Boston University School of Public Health, Boston, Massachusetts, United States

## Abstract

The pictorial superiority effect (PSE) is the finding that memory for pictures exceeds that of memory for matching words for people of all ages (Cherry et al., 2012). We examined free recall of line drawings and matching words in adults enrolled in the LSU Flood Study, an interdisciplinary study of disaster stress and cognition. We tested the hypothesis that disaster stress would be associated with deficits in memory for pictures and words. Participants were sampled from a three-parish (county) region of Baton Rouge, LA that was severely devastated by the 2016 flood (N = 202, age range: 18-88 years). They received multiple tests, including the Montreal Cognitive Assessment (MoCA; Nasreddine et al., 2005), and self-report measures of executive function and functional impairment (Barkley, 2011). Three groups were compared: (1) non-flooded adults as controls, (2) once-flooded adults with structural damage to homes and property in 2016, and (3) twice-flooded adults who had relocated to Baton Rouge because of catastrophic losses in Hurricanes Katrina and Rita and flooded again in 2016. Results yielded a PSE in free recall for all disaster exposure groups (p < 0.001). Follow-up analyses by age group revealed that older adults showed the same memorial advantage of pictures relative to words as did their younger counterparts across all disaster exposure groups. These results imply that single and multiple disaster exposures do not appear to disrupt cognition assessed with traditional, laboratory-based measures. This research was supported by a grant from the National Science Foundation (Award Number 1708090).

